# Guiding developments in flood-prone areas: Challenges and opportunities in Dire Dawa city, Ethiopia

**DOI:** 10.4102/jamba.v11i3.704

**Published:** 2019-07-05

**Authors:** Juliet Akola, Joseph Binala, Jimmy Ochwo

**Affiliations:** 1 Department of Urban and Regional Planning, University of Venda, Thohoyandou, South Africa; 2 Institute of Urban Development Studies, Ethiopian Civil Service University, Addis Ababa, Ethiopia

**Keywords:** Floods, Developments, Kebele, Dire Dawa, Challenges, Ethiopia

## Abstract

One of the biggest challenges that developing countries are facing today is the management of development in flood-prone areas. Ethiopia is no exception, as it has suffered perennial floods in different parts of the country with devastating consequences. Floods in Dire Dawa are occurring more frequently than before and this is attributed to climate change, among other reasons. This study looks at guiding development in flood-prone areas in Dire Dawa. In this study, data were collected from selected kebeles (zones). Questionnaires, interviews and observation were used as data collection methods. Respondents were randomly selected from the communities for the administration of questionnaires. Analysis revealed that the return period of floods in Dire Dawa is getting shorter. The local authorities have come up with coping strategies, which are falling short of the expected outcome, with poor implementation being the major problem. The study concludes by recommending that the administration needs to identify and prioritise existing opportunities by implementing the already existing strategies. The city administration needs to establish a specific body for watershed management and flood protection responsible for carrying out research, early warnings, designs, pooling resources, implementing and managing interventions.

## Introduction

One of the biggest challenges that developing countries are facing today is how to plan and manage developments in flood-prone areas. Good planning is key to minimise long-term risks of damage from flooding. The principle is simple – plan and develop properties, buildings and structures so that they are safe from flooding from the outset without compromising the safety of other properties. Prevention is far cheaper than cure (Melbourne Water 2007). Poor planning has often led to enormous loss of valuable assets in the past. In many cases, the impossibly high costs of flood mitigation works far outweigh the benefits of better protection, and such areas are unlikely to ever be relieved of the threat of flooding (World Bank 2013).

Worldwide, planning system plays a major national and local role in ensuring that developments that take place in flood-prone areas are encouraged and guided in a way that is sustainable (Office of Public Works 2009).

Flooding is a worldwide experience that causes widespread economic damages and loss of human lives (World Bank 2014). Over the past five years, destructive floods have incurred in certain parts of the USA, Australia, Asia and Africa, induced by mudslides, earthquake, tsunami and hurricane (Bloch, R. et al 2012). The occurrence of floods is the most frequent among all natural disasters. The number of people affected by floods and financial, economic and insured damages have all increased. In 2010 alone, 178 million people were affected by floods and the total loss in exceptional years such as 1998 and 2010 went above US$40 000 billion (United City 2013).

Over the past 62 years, flooding has afflicted several areas of the eastern and southern Ethiopia, killing hundreds and displacing hundreds of thousands of people (BBC News 2006). It may be imperative to note that many approaches have been employed to deal with disaster risks elsewhere in the world, including Ethiopia. It is unbelievable that after a period of about 9 years from the time of 2006 floods, some of the affected communities were still living in camps (DDADPFSO 2014). This position portrays the unpreparedness of the Dire Dawa City Administration to handle such disasters in a short- and long-run period. Dire Dawa town being one of the most growing cities in Ethiopia has an Integrated Development Plan (IDP), which seeks to guide developments and regulate land use. Under the IDP, there is also a Sustainable Development Framework (SDF) which consists of guidelines on how to develop Dire Dawa City Administration, in particular, on existing land use, road networks, building height, storm water drainage and intervention action plans. However, with the existence of the development plans (IDP & SDF) mentioned above, Dire Dawa City Administration appears not to be prepared towards managing floods and yet it is one of the towns that is prone to natural disasters, like floods and drought (Fortune News Paper 2012). The frequency of floods in urban Dire Dawa, Ethiopia, indicates that the occurrence of floods has been increasing from 1945, 1977, 1981, 1997, 2001, 2004, 2005 to 2006. The main aim of this study was to assess guidance of developments in Dire Dawa town in Ethiopia, specifically on describing the severity of floods, determining the benefits, identifying the key challenges and determining the opportunities related to guiding developments in flood-prone areas.

## Related literature

### World wide severity of floods

The world is witnessing and experiencing increased severe flooding. According to the World Bank, natural disasters are increasing in frequency and intensity (World Bank 2014); for example, between 1901 and 1910, there were 82 recorded disasters, but between 2003 and 2012 there were more than 4 000 disasters. Of great distress is the incidences relating to climate change. It is evident that the situation of floods is increasing in northern hemisphere of Africa, this can be seen with the floods that occurred in 2007 whereby more than 1 million people were affected in about 20 countries, particularly in Uganda, Ethiopia, Sudan, Burkina Faso, Togo, Mali and Niger. In the southern hemisphere countries like Mozambique, Zambia, Zimbabwe, Botswana, Madagascar and South Africa, a devastating flood in 2000 left 1.25 million people homeless, hundreds of people dead and destroyed crops, livestock and major infrastructural facilities (BBC NEWS 2006).

### Benefits of guiding developments

Guiding developments in flood-prone areas, which relates to controlling and managing floods, can provide a range of benefits, such as making better city budgets, through being a strong marketing tool for attracting investors and contributing to public health and poverty eradication (Cities Alliance 2007).

Guiding developments helps to restore and maintain flood plains. As cities grow, human settlements in flood-prone cities expand to areas prone to flooding without due consideration of the flood risks involved; however, land use zoning as a tool and its proper enforcement tries to prevent such developments from going on (World Bank 2007). This is strengthened by the availability of strategic plans, actions plans, IDPs, the legal and institutional disaster preparedness framework, good governance principles, right from the national to local levels.

### Challenges of guiding developments

In the process of guiding developments in flood prone areas by local governments, there are challenges to guide developments in flood-prone areas, there are challenges emanating from informal settlements that cities and municipalities encounter, among which flooding is one (Bulkeley et al. 2009). The urban population is opting to live at the outskirts of the cities, which goes beyond the planning boundaries. In developing countries, the poor in urban areas tend to occupy and settle in unpleasant places, like valleys, near swamps and wetlands, and for this reason when flooding occurs they are easily vulnerable (Satterthwaite 2008).

## Opportunities of guiding developments

Studies conducted in developed and developing countries reveal that combinations of opportunities are vital in terms of shaping the capacity of guiding developments in flood-prone areas. These opportunities include national and a local framework within which planning is conducted. Planning frameworks (contingency plans and disaster preparedness plans) need to enable local authorities to address flooding within the planning system. For example, recent reforms to planning guidance in the United Kingdom now mandate local action for mitigation and adaptation, enabling planning authorities to take flooding and its impacts into account in their decision-making and reducing the likelihood of challenges to planning decisions or regulations from land developers and the building industry (IPCC 2007).

**FIGURE 1:**
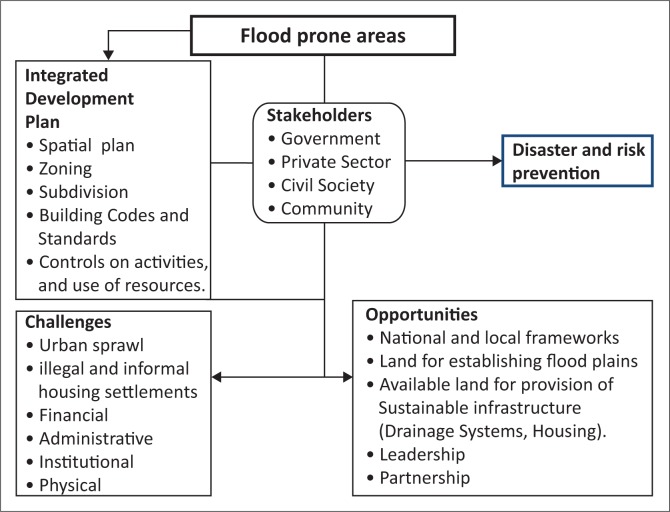
Conceptual framework of the study.

Figure 1 presents a conceptual framework related to guiding developments in flood-prone areas and how to overcome disasters related to flooding before, during and after the occurrence of floods.

Municipalities already have established strategies right from the international to local levels that can enable them to deal with such disasters. This study centers on the need to consider addressing challenges and more emphasis should be put on opportunities of guiding developments in flood-prone areas. Existence of an IDP of Dire Dawa is a great opportunity. It illustrates the various stakeholders in a comprehensive manner covering all, community, civil society and Non-governmental Organisations (NGOs), and the private sector.

Frequently a single tool in guiding developments does not operate in isolation. A mixture of diverse types of tools can be used together to prevent and minimise risks and disasters, for example, spatial planning, zoning of land use, building codes and standards. Some planning regulatory tools may have an effect in the long run, while others may work in the short run. The mixture of tools in guiding developments can continue over time to adapt to changing circumstances. Today, it is generally known that increasing the level of stakeholder participation in guiding developments leads to greater focus, importance and improves execution.

## Research method and design

**FIGURE 2:**
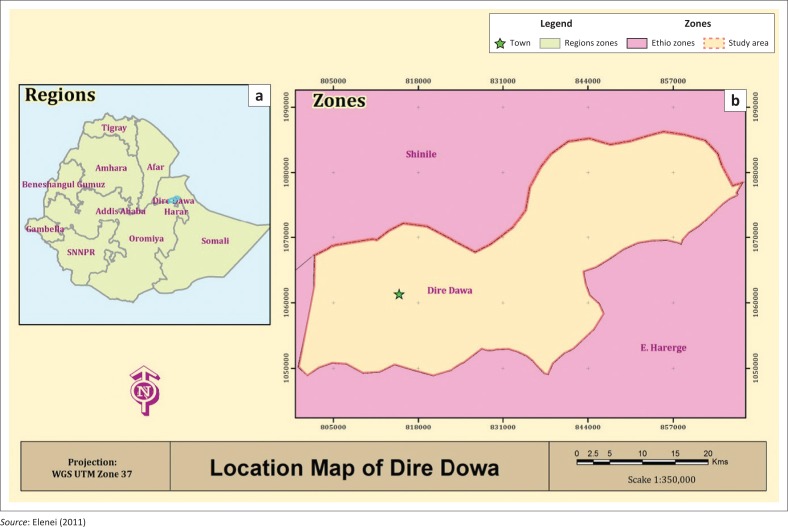
(a & b) Location of Dire Dawa city.

Geographically, this study was restricted to Dire Dawa City, covering 1213 km^2^, with an elevation of 5604 ft and holding an estimated population of 233 224 people (CSA 2007), of which 116 232 are men and 116 992 are women (see Table 1). Conceptually, the study was confined to describing the severity of floods, determining the benefits of guiding developments in flood-prone areas, identifying the key challenges of guiding developments in flood-prone areas and determining the opportunities related to guiding developments in flood-prone areas. Dire Dawa lies in the eastern part of Ethiopia, on the Dechatu River, at the foot of a ring of cliffs, with a latitude and longitude of 9°35’3502”N 41°51’58.00”E, coordinates. It is the second largest city administration in Ethiopia as indicated in Figure 2.

**TABLE 1:** Population and households for each kebele in Dire Dawa.

Kebeles	Both sex	Male	Female	Number of households	Estimated housing units
01	11 886	5958	5938	2115	-
02	49 428	24 550	24 878	13 366	-
03	19 884	10 077	9807	5540	55.4
04	21 047	10 118	10 929	5993	-
05	17 938	8624	9314	5445	54.45
06	16 671	8184	8487	4147	41.47
07	23 002	11 564	11 438	3963	39.63
08	29 638	14 370	15 268	5756	-
09	43 730	22 787	20 942	8250	-

**Total**	**233 224**	**116 232**	**117 001**	**54 575**	**190.95**

*Source*: Adapted from Dire Dawa City Administration (2014)

A descriptive case study approach was employed in which challenges and opportunities of guiding developments in Dire Dawa City were assessed, involving a description of what happened over the past and currently relating to guiding developments without changing the environment. This research approach enabled the researchers to obtain information on what happened with the 2006 floods, in particular, the current status, behaviour, attitudes and other characteristics with regard to flood risk and disasters among the people of Dire Dawa disaster-prone kebeles. Bickman and Rog (1998) suggest that descriptive studies can answer questions such as ‘what is’ or ‘what was’ – the questions this study seeks to answer with regard to guiding developments in flood-prone areas.

One hundred respondents for this study were randomly selected from the communities that live in flood-prone areas. They helped to describe the current situation of floods, including the history of what transpired during the 2006 flash floods. Purposively, 28 key informants from the flood-prone kebeles, concerned ministries such as the Ministry of Works and Urban Development, Environment and Beautification Agency and disaster prevention agencies were selected and included in the study as they are the officials who deal with planning and managing disasters. Community elders were considered too based on the time lived in Dire Dawa. Four kebeles from a list of nine villages were purposively selected for this study based on the aspect of flood susceptibility, vulnerability because of location and past history of flood hazards (see Table 2). The random sample was objectively drawn from a list of four villages provided by the Dire Dawa City Risk and Disaster Prevention Office.

**TABLE 2:** A description of the selected kebeles that are susceptible to flooding in Dire Dawa.

No	Name of kebeles	Name of specific areas under each kebele	Flooding-related information	
			Highly flood-prone areas within each kebele	Severity of floods
1	01 Kebele	Melka Jebdu	Not prone	Not prone
2	02 Kebele	Sabiyan, Goro, Genede Tesfa,	Sabiyan, Goro	Moderate
3	03 Kebele	Kezira, Nemberwan, Sefere Selam	Sefere Selam	Very high
4	04 Kebele	Meberat Hayel, Genede Kore, Gerikamp	Meberat Hayel (Genede Boye)	Moderate
5	05 Kebele	Addis Ketema, Genefele, Koka,	Addis Ketema, Genefele, Koka	Very high
6	06 Kebele	Dechatu, Kefira, Konel	Dechatu,	Very high
7	07 Kebele	Tayewan, Ashewa, Afete Issa,	Tayewan, Ashewa	Very high
8	08 Kebele	Legehare, Amestenga	Not prone	Not prone
9	09 Kebele	Police Meret, Genede	Not prone	Not prone

*Source*: DDPFSO (2014)

In order to determine the actual sample size, estimated housing units were considered on the following assumptions that Dire Dawa flood-prone kebeles are now sparsely populated, and people have migrated to safer kebeles. The majority of the population living in Dire Dawa are Muslims who marry more than one wife and have an average number of seven children and above and therefore an average of 10 people per household were considered. This aided in determining the housing units for the four kebeles purposively chosen for the study. Using the Kothari (2004) formula, the actual sample size for the study was 128 as follows: *n* = z2pq/d2, *n* = (1.96)2 (0.25)/0.025 = 384., Fn = n/(1+n/N), fn = 384/(1+384/190.95) = fn = 128 (where *n* is the desired sample size, *z* is the standard normal variable at a required level of confidence, *p* is the proportion in the target population estimated to have characteristics being measured, *q* = 1-p and *d* is the level of statistical significance set).

Descriptive statics in form of frequencies, percentages and ratios were generated to analyse quantitative data. Content analysis was used for qualitative data.

## Results

### Severity of floods in Dire Dawa

There are two major types of flood events that regularly occur in Dire Dawa. The first type of flood takes place during torrential rains because of mountain runoff in upper catchment of Kersa and Haramaya Wereda as well as because of localised heavy rainfall in Dire Dawa when high levels of water overflow the Dechatu and Goro rivers. This type of flood occurs mainly during the rainy season around April and May and around late July and early August. These floods were reported in 1997, 2001, 2003 and 2006. The other type is flash flooding, which occurs from heavy localized rainfall. These floods were reported in 1981, 1988, 1977, 2003 and 2004. Dire Dawa floods are favored by its topography, land cover, runoff from highland and intensive rainfall condition. Flash floods with a value of above 12.5 mm/h can cause high peak discharge, capable of inflicting hazards depending on the type of topography, soil property, effect of rainfall over upstream establishing the occurrence of floods over flood-sensitive areas.

**FIGURE 3:**
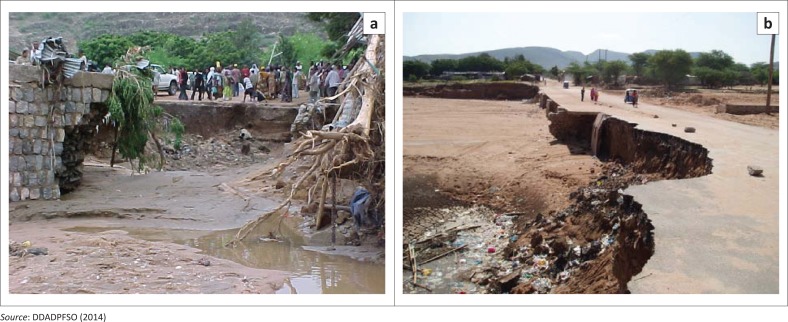
(a & b) Damage caused by 2006 floods on Dachatu Bridge and the road.

Results from Risk and Disaster Prevention officials confirm that, indeed, floods are prone in Dire Dawa city because of a number of factors which are manmade and natural (DDADPFSO 2014). The latest incident of flooding happened in August 2006 when Dechatu River overflowed its banks whereby an estimate of over 200 people were reported dead, thousands were displaced and there was extensive damage to homes and markets. Floods in Dire Dawa are fairly common during June to September, which is the rainy season. Based on the draft report by Adam et al. (2006:10), the devastating flood that occurred on 06 August 2006 caused the death of about 256 people, displaced 9956 people and 244 people went missing. The flood caused huge damage to urban infrastructure, roads, bridges and houses. Investors, traders, small and petty traders lost property worth Ethiopian Birr 30 054 275 (US$3 756 784.38). Figure 3 illustrates the damage caused by the 2006 floods on existing infrastructure.

### Guiding developments benefits in Dire Dawa

**FIGURE 4:**
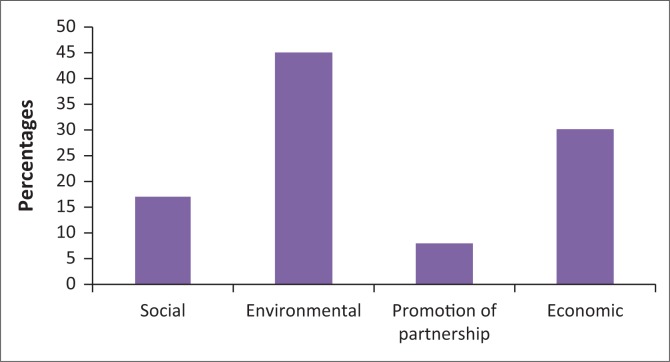
Benefits of guiding developments in flood-prone areas.

The Ministry of Works and Urban officials explained that the overall aim of guiding developments in flood-prone areas is to achieve orderly, coordinated, efficient and environmentally sound, social and economic development, and to secure the proper use of land. These benefits were also further pointed out by the communities in various kebeles, where 45% of the community explained that once developments are guided spatially through segregation of land use, enforcement of building codes and standards, then there is an environmental benefit, while 30% indicated that it saves economically from losses (see Figure 4) which shows the benefits indicated by the community in Dire Dawa city.

The environmental benefit here is that the community has less damage to the natural environment and the existing ecosystem. The city administration has embarked on an environmental campaign whereby the urban settlers are being monitored through setting river bank reserves. The farmers together with developers have to follow the set reserve standards to avoid damaging the river banks.

Twenty-five percent of the community also indicated that the people who lost their lives during the 2006 disaster were not informed of the consequences of settling in ecologically sensitive areas, whereas 34% did not have a choice but to settle in such areas. Because of efforts of the city administration, the current population living near the rivers, forests and valleys are being sensitised on the need of proper regulation guidelines and this is done through community head leaders on climate change in Dire Dawa.

Socially, the benefit is that the poor will not be displaced and loss of lives will be avoided. Socially, 17% of the community wished if their relatives were still alive for purposes of benefiting from social capital. There are still three camps that exist in Dire Dawa City Administration as a result of the 2006 floods. Socially and psychologically the community living in the three camps of Jerba, Gende and Marriam are finding it difficult to cope with the situation because food is not adequate, rising cost of living affects household expenditure, housing is not very convenient as the city administration cannot provide all the social services. The IDP emphasises on the availability of relatively safe housing in safe sites for all urban dwellers.

Economically, Dire Dawa City Administration experts explained that once developments are guided, actions that encompass all areas of a city and all sectors of a society, such as provision of infrastructure in planned areas and proper drainage channels for all areas, are monitored. Floods come with damage to property, lives and livelihood. Therefore, well-provided housing, infrastructure and maintenance will help to avoid unnecessary expenses when flood hazards occur.

### Promotion of partnership in addressing any risks and disasters

This has helped the Dire Dawa community to achieve good governance practices and innovative ideas from both the international and local communities, for example, through investment to make the whole city relatively safe; and the provision by local government of an enabling environment for local civil society action to help address and implement the aims in the participative IDP process.

### Guiding development challenges in Dire Dawa

The officials working in the disaster and prevention and food security office singled out in particular financial capacity of the administration to plan and prepare for any catastrophic event. The concerned officials explained that there is inadequate flow of funds to support the implementation of planned activities in the sector. Because of the budgetary constraints, there is hardly any money for the department to create awareness on flood risks and then take precaution on the practices of farming (IDP 2012).

**FIGURE 5:**
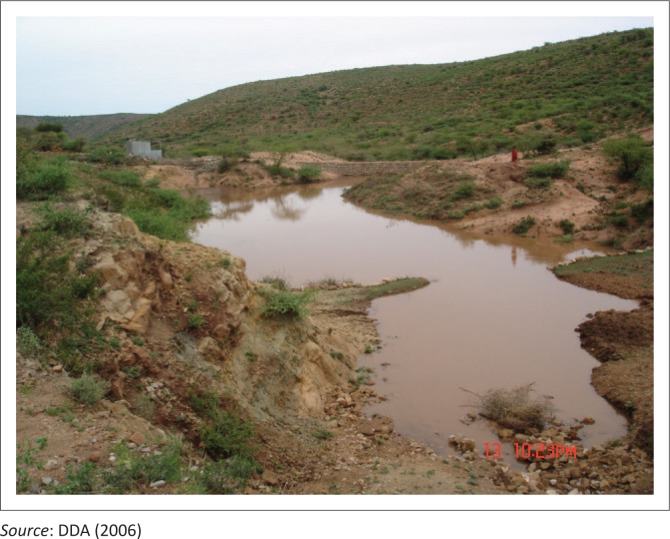
Part of the steep slopes and rugged topography.

Dire Dawa’s physical location is situated at the foot of the eastern highlands escarpment, crossed by four rivers through or around the urban area. At least two of the rivers, that is, Dechatu (catchment area 157 km^2^) and Goro (catchment area 83 km^2^), present a severe flood risk to the city’s infrastructure and inhabitants, commonly attributed to the state of the upstream catchments (see Figure 5).

Poor institutional capacity is yet another challenge that the administration faces in trying to guide developments in Dire Dawa. During the peak rainy seasons in April and August, the capacity of officials assigned to monitor the flood situation is not adequate. The office requires more staff as opposed to two staff experts currently running the activities of the office. In addition, because of budgetary constraints the officials are not in a position to carry out activities as a team and this affects monitoring and evaluation of the flood risks.

Weak implementation of urban plans is also a challenge faced by Dire Dawa City Administration. Like any other city administration, has got an IDP which is relevant technically, institutionally and has a policy framework with which the active participation of the relevant urban actors systematically identify, prioritise and address socio-economic, environmental and spatial urban development issues. It outlines intervention approaches and implementation programmes to be undertaken to achieve city-wide developments. It is one of the basic urban management tools, which professionals involved in urban development planning and implementation use in their daily activities including guiding developments to avoid future incidences of flood hazards.

### Opportunities of guiding developments

A set of circumstances exists to enable Dire Dawa Administration to positively guide developments in its flood-prone areas.

Committed leadership has been identified as a key factor shaping local capacity to act on disaster prevention through officials as individual leaders within the administration. The Ethiopian government, from 2007 to date, has frequently monitored the country’s disasters through policies, programmes and strategies. The latest one is the Growth Transformation Plan, which is aimed to guide all sectors of the economy for the next five years. Through the transformation plan, Dire Dawa City Administration together with the central government can establish regional disaster risk reduction committee leaders at community level, specifically from kebeles that are prone to flooding.

The existence of the Town Plan is an opportunity in itself as most towns in Ethiopia do not have development plans. A five-year IDP (2006–2011) for Dire Dawa Administration and a 10 year Spatial Development Framework (2006–2016) were prepared under the Dire Dawa Integrated Development Plan by the Federal Urban Planning Institute in July 2006. The Spatial Development Framework is intended to serve as an instrument in guiding the development of the city during the next 10 years and facilitate the preparation of detailed plans or local development plans without which the implementation may not be feasible. Different local development plans were prepared for Kezira, Megala, Boren and Goro areas of the town by Wondimu Consult.

The low population of 233 224 people makes it an opportunity for Dire Dawa to still implement the structural plan with no difficulties, the north eastern part of Dire Dawa is a sparsely populated lowland exhibiting agro-pastoral and pastoral system, and the southeastern part of the administration comprises the escarpment with mixed farming system. The low population enables the government to have enough land to gazette buffer zones such that trees and grass can be planted to help control surface runoffs during the rainy season.

### International support

Ethiopia being the seat of African Union and harboring the Economic Commission for Africa has attracted enormous attention in various sectors, including disaster prevention. Officials indicated that ever since 2006, the international community has tried to intervene through community sensitisation programmes whereby the awareness is created on how to avoid such disasters like floods. Also capacity building programmes have been carried out and institutions are encouraged to enroll students in climate change-related courses whereby disaster prevention is given attention; for example, Ethiopian Civil Service University, Mekelle University and Addis Ababa University have such programmes dealing with climate change (Ministry of works and Housing Development, 2005; Ministry of Finance and Economic Development, 2010).

### Ethical consideration

The principles of ethics and systematic research process were considered in this study to avoid harming human beings, animals and the natural environment of Dire Dawa City Administration flood prone areas. Babbie’s (2015) fundamental rules of social research were followed in this study. Participation principle of ethics was used to obtain consent and voluntary opinions of the research subjects. The aspects of privacy, anonymity and confidentially were highly considered in the process of conducting interviews and administering questionnaires. In order to ensure confidentiality, all documents containing research information were password protected. Anonymity was ensured by making sure that no identity was revealed for all participants in the study. In addition, the Ethiopian Civil Service University ethical clearance certificate and permission to conduct research from Dire Dawa City Administration were obtained and consent forms to respondents explaining the purpose of the study were prepared. Participants’ permission to record videos and audio was also sought. Finally, all findings of the study can be publically accessed from the online repository.

## Recommendations

The study recommends that given the vast opportunities in Dire Dawa, the city administration needs to identify and prioritise its opportunities by strengthening and implementing the already existing strategies. At an integrated level, the city administration may need to establish a specific body for watershed management and flood protection responsible of carrying out research, early warnings, designs, pooling resources, implementing and managing the interventions, collaborating with neighboring regions and involving NGOs in such disaster prevention programmes and projects. Currently there is an office on disaster prevention and food security. This office needs to be strengthened further through staffing and provision of adequate funds to spearhead the integration of activities together with the urban planning department.

## Conclusion

Flooding occurs in Dire Dawa city because of natural and manmade causes. This study aimed at assessing the challenges and opportunities of guiding developments in flood-prone areas in Dire Dawa Administration. The results of the study indicate that Dire Dawa experiences two rainy seasons associated with two flood regimes throughout every year. However, the guidance of developments through an IDP in flood-prone areas has made Dire Dawa witness less property damages and loss of lives in 2010 with the flood that was minor. There are social, economic and environmental benefits attached to guiding developments in the city. Amidst the benefits, the city is faced with challenges like inadequate financial resources, among others, which is very crucial in terms of guiding developments. In spite of all the efforts carried out by the city administration, the existing opportunities need to be considered in trying to manage disasters. There is still adequate land to enable the city administration to plan for proper infrastructure and flood plains. This article suggests an increased integrated approach among stakeholders towards addressing challenges and identifying the existing opportunities, such as the leadership commitment, location, international collaboration and support, to be able to plan and guide developments towards flood risk prevention.
